# PET / MR as tool for ”precision medicine”

**DOI:** 10.1186/1470-7330-14-S1-O34

**Published:** 2014-10-09

**Authors:** Markus Schwaiger

**Affiliations:** 1 Department of Nuclear Medicine, Klinikum rechts der Isar, Technische Universität München, Ismaninger Straße 22, 81675 München, Germany

## 

With the advent of multimodality imaging, molecular information by tracer techniques can be combined with high resolution structural and functional imaging by MRI. Specific tracers address biological processes of the oncogenic process, which allow not only early detection of malignant tissue, but may also provide prognostic information of oncological diseases. Most importantly, imaging biomarkers can be used to select patients for a given therapy (companion diagnostics) and to monitor response in order to optimize therapeutic strategies tailored to the needs of the individual patient. Examples of new molecular tracers are Gallium-68 labeled peptides targeting integrins, ligands of the chemokine CXCR4 and somatostatine receptors. These tracers do not only address cell surface proteins (receptors), but also the interaction of cells as well as cell migration, which have been linked to angiogenesis and metastasis. Since FDG is not very useful in patients with prostate cancer, the recent introduction of Gallium-68-PSMA has added excellent diagnostic sensitivity and specificity for PET diagnosis of prostate cancer, especially in patients with recurrent disease. The combination of molecular targeting of prostate cancer with measurements of regional perfusion (DCE) and cellularity (DWI) provided by MRI offers a comprehensive and innovative multimodality diagnostic approach. Regional phenotyping of malignant tissue addresses the spatial as well as dynamic heterogeneity of cancer, which may provide unique insights into mechanisms of therapeutic response and resistance. It can hypothesized, that multimodality molecular imaging and radionuclide therapy will be an important part of the emerging concepts of “theranostics” and further strengthen the role of Nuclear Medicine in precision medicine evaluating cancer patients.

